# Predisposition of HLA-DRB1*04:01/*15 heterozygous genotypes to Japanese mixed connective tissue disease

**DOI:** 10.1038/s41598-022-14116-x

**Published:** 2022-06-15

**Authors:** Shomi Oka, Takashi Higuchi, Hiroshi Furukawa, Kota Shimada, Atsushi Hashimoto, Akiko Komiya, Toshihiro Matsui, Naoshi Fukui, Eiichi Suematsu, Shigeru Ohno, Hajime Kono, Masao Katayama, Shouhei Nagaoka, Kiyoshi Migita, Shigeto Tohma

**Affiliations:** 1 Department of Clinical Research, National Hospital Organization Tokyo National Hospital, 3-1-1 Takeoka, Kiyose, 204-8585 Japan; 2 Clinical Research Center for Allergy and Rheumatology, National Hospital Organization Sagamihara National Hospital, 18-1 Sakuradai, Minami-ku, Sagamihara, 252-0392 Japan; 3 Department of Nephrology, Ushiku Aiwa General Hospital, 896 Shishiko-cho, Ushiku, 300-1296 Japan; 4 Department of Rheumatology, National Hospital Organization Sagamihara National Hospital, 18-1 Sakuradai, Minami-ku, Sagamihara, 252-0392 Japan; 5 Department of Rheumatic Diseases, Tokyo Metropolitan Tama Medical Center, 2-8-29 Musashi-dai, Fuchu, 183-8524 Japan; 6 Department of Clinical Laboratory, National Hospital Organization Sagamihara National Hospital, 18-1 Sakuradai, Minami-ku, Sagamihara, 252-0392 Japan; 7 Department of Internal Medicine and Rheumatology, Clinical Research Institute, National Hospital Organization Kyushu Medical Center, 1-8-1 Jigyohama, Chuo-ku, Fukuoka, 810-8563 Japan; 8 Department of Rheumatology, Shin Koga Hospital, 120 Tenjin-machi, Kurume, 830-8577 Japan; 9 Center for Rheumatic Diseases, Yokohama City University Medical Center, 4-57 Urafune-cho, Minami-ku, Yokohama, 232-0024 Japan; 10 Department of Internal Medicine, Teikyo University, 2-11-1 Kaga, Itabashi-ku, Tokyo, 173-8605 Japan; 11 Department of Internal Medicine, National Hospital Organization Nagoya Medical Center, 4-1-1 Sannomaru, Naka-ku, Nagoya, 460-0001 Japan; 12 Department of Rheumatology, Yokohama Minami Kyosai Hospital, 1-21-1 Rokuura-higashi, Kanazawa-ku, Yokohama, 236-0037 Japan; 13 Clinical Research Center, National Hospital Organization Nagasaki Medical Center, 2-1001-1 Kubara, Omura, 856-8562 Japan; 14 Department of Gastroenterology and Rheumatology, Fukushima Medical University School of Medicine, 1 Hikarigaoka, Fukushima, 960-1295 Japan; 15 Department of Rheumatology, National Hospital Organization Tokyo National Hospital, 3-1-1 Takeoka, Kiyose, 204-8585 Japan

**Keywords:** Genetics, Immunology, Rheumatology

## Abstract

Mixed connective tissue disease (MCTD) is a rare systemic autoimmune disease characterized by the production of anti-U1 ribonucleoprotein antibodies and systemic symptoms similar to those of some other autoimmune diseases. *HLA-DRB1* polymorphisms are important genetic risk factors for MCTD, but precise associations of *DRB1* genotypes with MCTD have not been reported in Japanese people. Genotyping of *HLA-DRB1* and *-DQB1* was performed in Japanese MCTD patients (n = 116) and controls (n = 413). Associations of specific allele carriers and genotype frequencies with MCTD were analyzed.The following alleles were found to be associated with predisposition to MCTD: HLA-DRB1*04:01 (*P* = 8.66 × 10^–6^, *Pc* = 0.0003, odds ratio [OR] 7.96, 95% confidence interval [CI] 3.13‒20.24) and DRB1*09:01 (*P* = 0.0189, *Pc* = 0.5468, OR 1.73, 95% CI 1.12‒2.67). In contrast, the carrier frequency of the DRB1*13:02 allele (*P* = 0.0032, *Pc* = 0.0929, OR 0.28, 95% CI 0.11‒0.72) was lower in MCTD patients than in controls. The frequencies of heterozygosity for HLA-DRB1*04:01/*15 (*P* = 1.88 × 10^–7^, OR 81.54, 95% CI 4.74‒1402.63) and DRB1*09:01/*15 (*P* = 0.0061, OR 2.94, 95% CI 1.38‒6.25) were also higher in MCTD patients. Haplotype and logistic regression analyses suggested a predisposing role for HLA-DRB1*04:01, DQB1*03:03, and a protective role for DRB1*13:02. Increased frequencies of HLA-DRB1*04:01/*15 and DRB1*09:01/*15 heterozygous genotypes were found in Japanese MCTD patients.

## Introduction

Mixed connective tissue disease (MCTD) is a rare systemic autoimmune disease characterized by the production of anti-U1 ribonucleoprotein (RNP) antibodies and the symptoms of several other systemic autoimmune diseases, including systemic lupus erythematosus (SLE), systemic sclerosis (SSc), rheumatoid arthritis, idiopathic inflammatory myopathy with Raynaud’s phenomenon, diffuse hand edema, polyarthritis, myositis, pleuritis, pericarditis, leukopenia, esophageal dysmotility, interstitial lung disease, and pulmonary hypertension^1–3^. A recent study reported the prevalence and the incidence of MCTD in European populations to be 3.8 per 100,000 and 2.1 per million per year, respectively^4^. In 2012, about 10,000 MCTD patients were reported to reside in Japan (https://www.mhlw.go.jp/file/06-Seisakujouhou-10900000-Kenkoukyoku/0000089901.pdf). The concept of MCTD as a discrete disease was originally proposed by Sharp^5^. However, there has been controversy as to whether a distinct MCTD clinical entity does exist, due to the similarity to other systemic autoimmune diseases^6–8^. The identification of a specific serological marker (anti-U1RNP antibodies) and the unique association of MCTD with the presence of *HLA*-DRB1*04 support the concept of MCTD as a discrete pathological entity^9,10^.

The etiology of MCTD is unknown. It is affected by genetic and environmental factors, with *HLA-DRB1* being an important genetic risk factor^1–3^. Several studies have reported that *HLA-DRB1* alleles are associated with MCTD, notably in an increased frequency of DRB1*01 and DRB1*04^11–17^. In one recent study it was found that HLA-DRB1*04:01, DRB1*09:01, and DRB1*15:01 were associated with susceptibility to MCTD in European populations^18^. Conversely, HLA-DRB1*04:04, DRB1*07:01, DRB1*13:01, and DRB1*13:02 were associated with protection against MCTD^17,18^. HLA-DRB1*04:01 and DRB1*09:01 were also associated with susceptibility to MCTD in studies of Japanese populations^19,20^. However, few studies of *HLA* in MCTD have considered populations other than European. It was also proposed that HLA-DRB1*04 is primarily associated with the production of anti-U1RNP antibodies^11,21,22^. The roles of these *DRB1* alleles have been analyzed in cohorts of modest size, and the effects of specific amino acid residues in the DRβ chain were not established. No assessments were made of any gene dosage effect of the predisposing alleles (i.e., homozygosity for the risk alleles conferring a higher risk than heterozygosity). The genotypes of these risk alleles were not precisely analyzed; the resolution of the genotyping methods in earlier studies was lower than is now attainable. Additionally, no genome-wide association studies have been reported for MCTD. Thus, the role of *HLA-DRB1* on susceptibility to MCTD has not been well studied due to its rarity. In this study we analyzed associations of *HLA-DRB1* genotypes with Japanese MCTD, using higher resolution genotyping methods.

## Results

### HLA-DRB1 and -DQB1 allele carrier frequency in MCTD

Genotyping of *HLA-DRB1* and -*DQB1* was conducted, to compare allele carrier frequencies in MCTD patients and controls (Table 1, Supplementary Table S1). (Allele carrier frequency is the frequency of individuals with a specific allele in some populations.) No deviation from the Hardy–Weinberg equilibrium was observed in the controls (*HLA-DRB1*: *P* = 0.4257; *DQB1*: *P* = 0.2162), but deviation was found in the MCTD patients (*DRB1*: *P* = 0.0130; *DQB1*: *P* = 0.2714). DRB1*04:01 was significantly associated with susceptibility to MCTD (*P* = 8.66 × 10^–6^, *Pc* = 0.0003, odds ratio [OR] 7.96, 95% confidence interval [CI] 3.13‒20.24). The association of HLA-DRB1*09:01 with predisposition to MCTD did not attain statistical significance after a Bonferroni correction (*P* = 0.0189, *Pc* = 0.5468, OR 1.73, 95% CI 1.12‒2.67). Neither did the association of HLA-DRB1*13:02 with protection against MCTD attain statistical significance after a Bonferroni correction (*P* = 0.0032, *Pc* = 0.0929, OR 0.28, 95% CI 0.11‒0.72). Tendencies were found for HLA-DQB1*03:03 (*P* = 0.0279, *P*c = 0.3903, OR 1.66, 95% CI 1.08‒2.57, Supplementary Table S2) to predispose to disease, and for DQB1*06:04 (*P* = 0.0150, *P*c = 0.2107, OR 0.33, 95% CI 0.13‒0.84, Supplementary Table S2) to protect against MCTD. Thus, higher allele carrier frequencies of HLA-DRB1*04:01 and DRB1*09:01, and a lower allele carrier frequency of DRB1*13:02, were found in MCTD patients than in controls.

**Table 1:** *HLA-DRB1* allele carrier frequencies in MCTD patients and controls.

	MCTD (n = 116)	Control (n = 413)	*P*	OR	*P*c	95% CI
DRB1*04:01	14 (12.1)	7 (1.7)	8.66 × 10^–6^	7.96	0.0003	(3.13–20.24)
DRB1*04:04	2 (1.7)	0 (0.0)	0.0478	18.06	> 1	(0.86–378.79)
DRB1*08:03	22 (19.0)	61 (14.8)	0.3114	1.35	> 1	(0.79–2.31)
DRB1*09:01	43 (37.1)	105 (25.4)	0.0189	1.73	0.5468	(1.12–2.67)
DRB1*13:02	5 (4.3)	57 (13.8)	0.0032	0.28	0.0929	(0.11–0.72)
DRB1*15:01	27 (23.3)	68 (16.5)	0.1008	1.54	> 1	(0.93–2.55)
DRB1*15:02	20 (17.2)	89 (21.5)	0.3637	0.76	> 1	(0.44–1.30)
*04:01, *09:01	57 (49.1)	112 (27.1)	1.51 × 10^–5^	2.60		(1.70–3.97)

Allele carrier frequencies are shown in parentheses (%). Association was tested by Fisher’s exact test, using 2 × 2 contingency tables. Allele carrier frequency is the frequency of individuals with a specific allele in some populations. MCTD: mixed connective tissue disease; OR: odds ratio; CI: confidence interval; *P*c: corrected *P* (*Pc* values greater than 1 are shown as “ > 1”).

### HLA-DRB1*04:01 allele carrier frequencies in MCTD associated with specific clinical characteristics

Associations of HLA-DRB1*04:01 with specific clinical characteristics of MCTD were then analyzed (Table 2). It was found to be significantly associated with susceptibility to: MCTD collagen vascular disease (*P* = 0.0013, *P*c = 0.0369, OR 9.35, 95% CI 2.81‒31.20); Raynaud’s phenomenon (*P* = 0.0003, *P*c = 0.0073, OR 8.00, 95% CI 2.80‒22.89); hand edema (*P* = 3.33 × 10^–5^, *P*c = 0.0010, OR 11.32, 95% CI 3.91‒32.80); and polyarthritis (*P* = 0.0008, *P*c = 0.0239, OR 8.49, 95% CI 2.72‒26.45). Thus, subsets of MCTD patients with certain clinical features exhibited markedly increased frequencies of HLA-DRB1*04:01.

**Table 2:** Association of HLA-DRB1*04:01 allele carrier frequencies with clinical characteristics of MCTD.

Phenotype	Number	DRB1*04:01( +)	*P*	OR	*P*c	95% CI
Male	12	1 (8.3)	0.2063	5.27	> 1	(0.60–46.61)
Age at onset < 45	40	4 (10.0)	0.0110	6.44	0.3181	(1.80–23.06)
Overlap of collagen vascular disease	36	5 (13.9)	0.0013	9.35	0.0369	(2.81–31.20)
Interstitial lung disease	48	5 (10.4)	0.0045	6.74	0.1263	(2.05–22.17)
Pulmonary arterial hypertension	18	2 (11.1)	0.0499	7.25	> 1	(1.39–37.71)
Raynaud's phenomenon	66	8 (12.1)	0.0003	8.00	0.0073	(2.80–22.89)
Esophageal involvement	29	3 (10.3)	0.0223	6.69	0.6481	(1.63–27.40)
Hand edema	49	8 (16.3)	3.33 × 10^–5^	11.32	0.0010	(3.91–32.80)
Polyarthritis	47	6 (12.8)	0.0008	8.49	0.0239	(2.72–26.45)
Muscle weakness	14	2 (14.3)	0.0315	9.67	0.9147	(1.81–51.51)
Controls	413	7 (1.7)				

DRB1*04:01 allele carrier frequencies are shown in parentheses (%). Association was tested by Fisher’s exact test, using 2 × 2 contingency tables. DRB1*04:01 allele carrier frequency is the frequency of individuals with a DRB1*04:01 allele in each population. Each MCTD subset with clinical characteristics was compared with controls. MCTD: mixed connective tissue disease; OR: odds ratio; CI: confidence interval; *P*c: corrected *P* (*Pc* values greater than 1 are shown as “ > 1”).

### HLA-DRB1 and -DQB1 genotype frequencies in MCTD

The *HLA-DRB1* genotype frequencies in the MCTD patients were compared with those in the controls (Table 3). (Genotype frequency is the frequency of individuals with a specific genotype in some populations.) Homozygosity for HLA-DRB1*09:01 was found to confer a higher risk of MCTD than heterozygosity (*09:01/not *09:01: *P* = 0.0666, OR 1.55, 95% CI 0.98‒2.44; *09:01/*09:01: *P* = 0.1523, OR 2.15, 95% CI 0.83‒5.58). This gene dosage effect was not observed for HLA-DRB1*04:01 or DRB1*13:01. Genotype frequencies of HLA-DRB1*04:01/DRB1*15:01 (*P* = 0.0022, OR 33.08, 95% CI 1.77‒619.03) and DRB1*04:01/DRB1*15:02 (*P* = 0.0001, OR 48.65, 95% CI 2.72‒870.21) were higher in MCTD patients than in controls. The frequency of HLA-DRB1*09:01/DRB1*15:01 (*P* = 0.0112, OR 3.75, 95% CI 1.38‒10.22) was also higher in MCTD patients, but that of DRB1*09:01/DRB1*15:02 was not. The frequencies of HLA-DRB1*04:01/*15 (*P* = 1.88 × 10^–7^, OR 81.54, 95% CI 4.74‒1402.63) and DRB1*09:01/*15 (*P* = 0.0061, OR 2.94, 95% CI 1.38‒6.25) were also higher in MCTD patients. Furthermore, the frequency of HLA-DRB1*04:01, *09:01/*15 (*P* = 3.45 × 10^–7^, OR 5.76, 95% CI 2.96‒11.22) in MCTD patients was higher than in the controls. Finally, the frequency of HLA-DQB1*03:03/DQB1*06:02 (*P* = 0.0178, OR 3.72, 95% CI 1.28‒10.85) was higher in MCTD patients than in controls (Supplementary Table S3). Thus, HLA-DRB1*04:01/DRB1*15 and DRB1*09:01/DRB1*15 heterozygous genotypes predispose to MCTD.

**Table 3:** *HLA-DRB1* genotype frequency in MCTD patients and controls.

	MCTD (n = 116)	Control (n = 413)	*P*	OR	95% CI
*04:01	14 (12.1)	7 (1.7)	8.66 × 10^–6^	7.96	(3.13–20.24)
*04:01/not *04:01	13 (11.2)	6 (1.5)	1.24 × 10^–5^	8.56	(3.18–23.07)
*04:01/*04:01	1 (0.9)	1 (0.2)	0.3908	3.58	(0.22–57.72)
*09:01	43 (37.1)	105 (25.4)	0.0189	1.73	(1.12–2.67)
*09:01/not *09:01	36 (31.0)	93 (22.5)	0.0666	1.55	(0.98–2.44)
*09:01/*09:01	7 (6.0)	12 (2.9)	0.1523	2.15	(0.83–5.58)
*13:02	5 (4.3)	57 (13.8)	0.0032	0.28	(0.11–0.72)
*13:02/not *13:02	5 (4.3)	52 (12.6)	0.0103	0.31	(0.12–0.80)
*13:02/*13:02	0 (0.0)	5 (1.2)	0.5909	0.32	(0.02–5.81)
*04:01/*13:02	0 (0.0)	1 (0.2)	1.0000	1.18	(0.05–29.17)
*09:01/*13:02	1 (0.9)	9 (2.2)	0.6987	0.39	(0.05–3.11)
*04:01/*15:01	4 (3.4)	0 (0.0)	0.0022	33.08	(1.77–619.03)
*09:01/*15:01	8 (6.9)	8 (1.9)	0.0112	3.75	(1.38–10.22)
*04:01/*15:02	6 (5.2)	0 (0.0)	0.0001	48.65	(2.72–870.21)
*09:01/*15:02	5 (4.3)	9 (2.2)	0.2017	2.02	(0.66–6.16)
*04:01/*15	10 (8.6)	0 (0.0)	1.88 × 10^–7^	81.54	(4.74–1402.63)
*09:01/*15	13 (11.2)	17 (4.1)	0.0061	2.94	(1.38–6.25)
*04:01, *09:01	57 (49.1)	112 (27.1)	1.51 × 10^–5^	2.60	(1.70–3.97)
*04:01, *09:01/*15:01	12 (10.3)	8 (1.9)	0.0002	5.84	(2.33–14.66)
*04:01, *09:01/*15:02	11 (9.5)	9 (2.2)	0.0010	4.70	(1.90–11.64)
*04:01, *09:01/*15	23 (19.8)	17 (4.1)	3.45 × 10^–7^	5.76	(2.96–11.22)

Genotype frequencies are shown in parentheses (%). Association was tested by Fisher’s exact test, using 2 × 2 contingency tables. Genotype frequency is the frequency of individuals with a specific genotype in some populations. Genotype frequency of the heterozygous genotype of DRB1*04:01 is shown in the row “*04:01/not *04:01” and that of the homozygous genotype of DRB1*04:01 is shown in the row “*04:01/*04:01”. MCTD: mixed connective tissue disease; OR: odds ratio; CI: confidence interval.

### HLA-DRB1-DQB1 haplotypes in MCTD

*HLA-DRB1-DQB1* haplotype carrier frequencies in MCTD patients were compared with controls (Table 4). (Haplotype carrier frequency is the frequency of individuals with a specific haplotype in some populations.) Higher haplotype carrier frequencies of HLA-DRB1*04:01-DQB1*03:01 (*P* = 2.88 × 10^–5^, OR 7.32, 95% CI 2.85‒18.81) and DRB1*09:01-DQB1*03:03 (*P* = 0.0090, OR 1.84, 95% CI 1.19‒2.86) and lower DRB1*13:02-DQB1*06:04 (*P* = 0.0050, OR 0.26, 95% CI 0.09‒0.73) were found in MCTD patients than in controls. However, no differences were seen for HLA-DRB1*09:01-DQB1*03:01 or DRB1*13:02-DQB1*06:09 haplotype carrier frequencies. These data suggest primary associations of HLA-DRB1*04:01, DQB1*03:03, and DRB1*13:02 with MCTD.

**Table 4:** *HLA-DRB1-DQB1* haplotype carrier or diplotype frequency in MCTD patients and controls.

	MCTD (n = 116)	Control (n = 413)	*P*	OR	95% CI
**DRB1-DQB1 haplotype**					
*04:01-*03:01	13 (11.2)	7 (1.7)	2.88 × 10^–5^	7.32	(2.85–18.81)
*09:01-*03:01	0 (0.0)	4 (1.0)	0.5809	0.39	(0.02–7.31)
*09:01-*03:03	43 (37.1)	100 (24.2)	0.0090	1.84	(1.19–2.86)
*13:02-*06:04	4 (3.4)	50 (12.1)	0.0050	0.26	(0.09–0.73)
*13:02-*06:09	1 (0.9)	6 (1.5)	1.0000	0.59	(0.07–4.95)
*15:01-*03:01	1 (0.9)	3 (0.7)	1.0000	1.19	(0.12–11.53)
*15:01-*06:02	25 (21.6)	65 (15.7)	0.1616	1.47	(0.88–2.46)
*15:02-*06:01	19 (16.4)	89 (21.5)	0.2429	0.71	(0.41–1.23)
**DRB1-DQB1 diplotype**					
*04:01-*03:01/*15:01-*06:02	3 (2.6)	0 (0.0)	0.0103	25.50	(1.31–497.34)
*09:01-*03:03/*15:01-*06:02	7 (6.0)	7 (1.7)	0.0178	3.72	(1.28–10.85)
*04:01-*03:01/*15:02-*06:01	6 (5.2)	0 (0.0)	0.0001	48.65	(2.72–870.21)
*09:01-*03:03/*15:02-*06:01	4 (3.4)	9 (2.2)	0.4953	1.60	(0.48–5.30)
*04:01-*03:01, *09:01-*03:03/*15:01-*06:02	10 (8.6)	7 (1.7)	0.0009	5.47	(2.03–14.71)
*04:01-*03:01, *09:01-*03:03/*15:02-*06:01	10 (8.6)	9 (2.2)	0.0027	4.23	(1.68–10.69)
*04:01-*03:01/*15:01-*06:02, *15:02-*06:01	9 (7.8)	0 (0.0)	9.13 × 10^–7^	73.08	(4.22–1265.70)
*09:01-*03:03/*15:01-*06:02, *15:02-*06:01	11 (9.5)	16 (3.9)	0.0281	2.60	(1.17–5.77)
*04:01-*03:01, *09:01-*03:03/*15:01-*06:02, *15:02-*06:01	20 (17.2)	16 (3.9)	4.97 × 10^–6^	5.17	(2.58–10.35)

Haplotype carrier or diplotype frequencies are shown in parentheses (%). Association was tested by Fisher’s exact test, using 2 × 2 contingency tables. Haplotype carrier frequency is the frequency of individuals with a specific haplotype in some populations. Diplotype frequency is the frequency of individuals with a specific diplotype in some populations. MCTD: mixed connective tissue disease; OR: odds ratio; CI: confidence interval.

*HLA-DRB1-DQB1* diplotype frequencies were also compared between MCTD patients and controls (Table 4). (Diplotype frequency is the frequency of individuals with a specific diplotype in some populations.) HLA-DRB1*04:01-DQB1*03:01/DRB1*15:01-DQB1*06:02 (*P* = 0.0103, OR 25.50, 95% CI 1.31‒497.34) and DRB1*04:01-DQB1*03:01/DRB1*15:02-DQB1*06:01 (*P* = 0.0001, OR 48.65, 95% CI 2.72‒870.21) conferred susceptibility to MCTD. The diplotype frequency of HLA-DRB1*09:01-DQB1*03:03/DRB1*15:01-DQB1*06:02 was higher in MCTD patients (*P* = 0.0178, OR 3.72, 95% CI 1.28‒10.85), but DRB1*09:01-DQB1*03:03/DRB1*15:02-DQB1*06:01 was not. Diplotype frequencies of HLA-DRB1*04:01-DQB1*03:01/DRB1*15:01-DQB1*06:02, DRB1*15:02-DQB1*06:01 (*P* = 9.13 × 10^–7^, OR 73.08, 95% CI 4.22‒1265.70), DRB1*09:01-DQB1*03:03/DRB1*15:01-DQB1*06:02, DRB1*15:02-DQB1*06:01 (*P* = 0.0281, OR 2.60, 95% CI 1.17‒5.77) were also higher in MCTD patients than in controls. Additionally, the frequency of HLA-DRB1*04:01–DQB1*03:01, DRB1*09:01–DQB1*03:03/DRB1*15:01–DQB1*06:02, DRB1*15:02–DQB1*06:01 was higher in MCTD patients (*P* = 4.97 × 10^–6^, OR 5.17, 95% CI 2.58‒10.35). Thus, some specific heterozygous diplotypes predispose to MCTD.

### Associations of amino acid residues in the HLA-DRβ chain with MCTD

**Figure 1:**
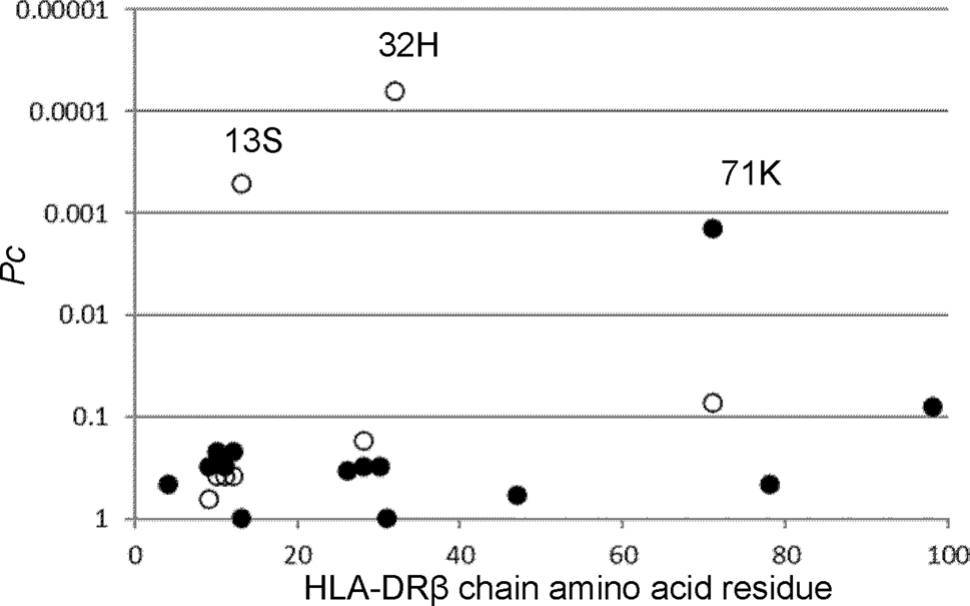
Associations of amino acid residues in the DRβ chain with MCTD. Significance of the associations was established by Fisher’s exact test, using 2 × 2 contingency tables. Corrected *P* (*P*c) values were generated by multiplying *P* values by the number of analyzed amino acid residues. Predisposing associations are indicated by filled circles and protective associations by open circles. MCTD: mixed connective tissue disease.

We next investigated whether the presence of any specific amino acid residues in the HLA-DRβ chain were associated with the occurrence of MCTD. Histidine at position 32 (32H, *P* = 1.83 × 10^–6^, OR 0.30, *P*c = 6.22 × 10^–5^, 95% CI 0.18‒0.51) and serine at position 13 (13S, *P* = 1.48 × 10^–5^, OR 0.33, *P*c = 0.0005, 95% CI 0.19‒0.56) were found to be associated with protection against MCTD (Fig. 1). Conversely, lysine at position 71 (71 K, *P* = 4.03 × 10^–5^, OR = 6.16, *P*c = 0.0014, 95% CI 2.59‒14.63) was associated with a predisposition to MCTD. Associations of amino acid residues in the HLA-DQβ chain were also analyzed (Supplementary Figure S1) but none was detected. Thus, certain amino acid residues in the HLA-DRβ chain but not in the HLA-DQβ chain were associated with susceptibility to or protection against MCTD.

### Logistic regression analysis of HLA alleles for MCTD

Because *DRB1* and -*DQB1* alleles are in strong linkage disequilibrium, a conditional logistic regression analysis between them in MCTD was performed to determine which locus was responsible for the observed associations (Supplementary Table S4). The association of HLA-DRB1*04:01 remained significant (*P*_adjusted_ = 4.49 × 10^–6^, OR_adjusted_ 12.46, 4.24‒36.62), when conditioned on DQB1*03:01. A protective association of HLA-DQB1*03:01 was detected (*P*_adjusted_ = 0.0106, OR_adjusted_ 0.44, 0.24‒0.83) when conditioned on DRB1*04:01, suggesting that the primary predisposing association for MTCD is with DRB1*04:01. The association of HLA-DRB1*09:01 was no longer apparent (*P*_adjusted_ = 0.8031, OR_adjusted_ 1.15, 0.39‒3.32) when conditioned on DQB1*03:03, but the association of DQB1*03:03 was still present when conditioned on DRB1*09:01, (*P*_adjusted_ = 0.5116, OR_adjusted_ 1.43, 0.49‒4.11), suggesting a primary role for DQB1*03:03. We also found that the protective association of HLA-DRB1*13:02 remained when conditioned on DQB1*06:04 (*P*_adjusted_ = 0.1522, OR_adjusted_ 0.16, 95% CI 0.01‒1.99), although it was no longer statistically significant. Finally, the protective association of HLA-DQB1*06:04 was no longer present when conditioned on DRB1*13:02 (*P*_adjusted_ = 0.5962, OR_adjusted_ 2.01, 0.15‒26.73), suggesting that the latter was the primary protective association. These suggested primary associated alleles in the logistic regression analysis were the same as those identified by haplotype analysis. Thus, certain *HLA-DRB1* or *DQB1* alleles seem to be primarily responsible for susceptibility to or protection against MCTD.

### HLA-DRB1*04:01allele carrier frequencies in SLE or SSc with anti-U1RNP antibodies

HLA-DRB1*04:01 allele carrier frequencies in SLE or SSc patients with anti-U1RNP antibodies were analyzed, to determine whether DRB1*04:01 was primarily associated with the production of anti-U1RNP antibodies or with susceptibility to MCTD (Supplementary Table S5). It is evident that HLA-DRB1*04:01 is associated with the production of anti-U1RNP antibodies in SLE. Thus, HLA-DRB1*04:01 may be associated with the production of anti-U1RNP antibodies in general.

## Discussion

In the present study we confirmed the associations of HLA-DRB1*04:01 and DRB1*09:01 with Japanese MCTD. DRB1*13:02 was also confirmed to be protective against MCTD.　The genotype frequencies of HLA-DRB1*04:01/DRB1*15 and DRB1*09:01/DRB1*15 were increased in MCTD patients. The predisposing role of HLA-DRB1*04:01, DQB1*03:03, and the protective role of DRB1*13:02, were suggested.

Previous studies have reported that HLA-DRB1*04:01, DRB1*09:01, and DRB1*15:01 are associated with susceptibility to MCTD^18^, and that DRB1*04:04, DRB1*07:01, DRB1*13:01, and DRB1*13:02 are associated with protection^17,18^ in European populations. HLA-DRB1*04:01 and DRB1*09:01 seem to be the common predisposing alleles in both European and Japanese populations, and DRB1*04:05 was a protective allele, at least in Japanese^19,20^. However, HLA-DRB1*15:01 was not found to be predisposing, and neither DRB1*04:04 nor DRB1*04:05 were protective in this study. These data suggest that it is HLA-DRB1*13:02 that is protective against MCTD in both European and Japanese populations, and that it is also the protective allele for multiple other systemic autoimmune diseases^23^.

The susceptibility alleles for MCTD were distinct from those for SLE (HLA-DRB1*15:01)^24^, SSc (DRB1*10:01)^25^, or idiopathic inflammatory myopathy (DRB1*08:03)^26^ in the Japanese. The susceptibility alleles for MCTD were similar to those for rheumatoid arthritis (HLA-DRB1*01:01, DRB1*04:01, DRB1*04:05, DRB1*04:10, DRB1*09:01, DRB1*10:01)^27^, except that DRB1*04:05 is the most important risk allele for Japanese rheumatoid arthritis, which was not shared with MCTD. Thus, different *DRB1* alleles were associated with susceptibility to or protection against MCTD, when compared with other collagen vascular diseases including SLE, SSc, idiopathic inflammatory myopathy, and rheumatoid arthritis. This supports the notion that MCTD is a distinct disease entity. A gene dosage effect was detected in the associations between *HLA-DRB1* risk alleles and rheumatoid arthritis^27^.

In the present study on MCTD, no gene dosage effect was found for HLA-DRB1*04:01 but it was found for DRB1*09:01, suggesting differential roles for DRB1*04:01 and DRB1*09:01 in the pathogenesis of MCTD.

Type 1 diabetes is an organ-specific autoimmune disease that affects pancreatic β cells; the frequencies of HLA-DRB1*03/DRB1*04 and DRB1*04/DRB1*08 heterozygous genotypes are increased in this disease^28^. HLA-DRB1*04/DRB1*08 heterozygous genotype frequencies are also increased in autoimmune hepatitis, another organ-specific autoimmune disease^29^. Analogously, the frequencies of HLA-DRB1*04:01/DRB1*15 and DRB1*09:01/DRB1*15 heterozygous genotypes are increased in Japanese MCTD, as found in the present study. Heterozygous genotypes of HLA-DRB1*04:01/DRB1*15 and DRB1*09:01/DRB1*15 might increase the probability of presentation of self-antigens and increase the risk of MCTD. Thus, the manner in which *DRB1* is associated with MCTD seems similar to type 1 diabetes and autoimmune hepatitis. The frequencies of heterozygous genotypes HLA-DRB1*03/DRB1*04 and DRB1*04/DRB1*08 were reported to be increased in type 1 diabetes, and it was proposed that *HLA-DRB1*, -*DQA1*, and -*DQB1* loci independently contributed to susceptibility to this disease^28^. These heterozygous risk genotypes were explained by *trans*-complementing DQα-β heterodimer molecules encoded by the *HLA-DQA1* allele of one haplotype and the *DQB1* allele of the other haplotype^28^. The frequencies of HLA-DRB1*04:01/*15 and DRB1*09:01/*15 heterozygous genotypes were also increased in Japanese MCTD, as shown here. HLA-DRB1*04:01 would be responsible for predisposition to MCTD in the DRB1*04:01-DQB1*03:01 haplotype and DQB1*03:03 in the DRB1*09:01-DQB1*03:03 haplotype, based on the data obtained from haplotype and logistic regression analyses in the present study. These data analogously suggest pathogenic roles of *trans*-complementing DQα-β heterodimer molecules in MCTD. *HLA-DRB1-DQB1* diplotype analyses showed that DRB1*09:01-DQB1*03:03/DRB1*15:01-DQB1*06:02 was associated with susceptibility to MCTD. The *HLA-DQA1* allele in the DRB1*09:01-DQB1*03:03 haplotype is likely to be DQA1*01:01, given haplotype distributions in Japanese^30^. However, the *DQA1* allele in DRB1*15:01-DQB1*06:02 is DQA1*01:02. The risk diplotype HLA-DRB1*09:01-DQB1*03:03/DRB1*15:01-DQB1*06:02 would encode DQA1*01:01-DQB1*06:02 and DQA1*01:02-DQB1*03:03 molecules in *trans*. Other culprit genes in linkage disequilibrium with *HLA-DRB1-DQB1* loci could also cause MCTD. Thus, several explanations can be proposed for the mechanisms of MCTD pathogenesis, based on results of analyses of associations with *HLA-DRB1* and -*DQB1*.

The present study found associations of amino acid residues 13S, 32H, and 71 K in the HLA-DRβ chain with MCTD. The 13S and 32H residues are encoded by HLA-DRB1*13:02, and 71 K by DRB1*04:01. The strongest protective association of 32H amino acid residue would reflect the protective effects of HLA-DRB1*13:02.

There has been some controversy as to whether DRB1*04 is primarily associated with the production of anti-U1RNP antibodies^11,21,22^. In the present study, HLA-DRB1*04:01 was associated with the production of anti-U1RNP antibodies in SLE, suggesting that DRB1*04:01 in MCTD was primarily associated with the production of anti-U1RNP antibodies, not for the pathogenesis.

In the present study, the predisposing association of HLA-DRB1*04:01 and DRB1*09:01 and the protective association of DRB1*13:02 with Japanese MCTD have been documented. To the best of our knowledge, this is the first report on associations of HLA-DRB1*04:01 and DRB1*09:01/DRB1*15 heterozygous genotypes with MCTD. HLA-DRB1*04:01-DQB1*03:01 and DRB1*09:01-DQB1*03:03 haplotypes were associated with MCTD. The association of HLA-DRB1*04:01-DQB1*03:01, DRB1*09:01-DQB1*03:03/DRB1*15:01-DQB1*06:02, DRB1*15:02-DQB1*06:01 diplotypes was also detected. It is suggested that specific combinations of *HLA-DRB1* and -*DQB1* alleles or haplotypes play important roles in the pathogenesis of MCTD.

The present study has some limitations. No replication analysis was conducted. The sample size was modest, although it is the largest in an Asian population. The distribution pattern of *HLA* alleles would differ in other ethnic populations. Other loci in the *HLA* region might contribute to the pathogenesis of MCTD. Thus, larger-scale studies of the *HLA* region in multiethnic populations should be performed to confirm the findings of the present study, despite the obstacle of the rarity of MCTD.

## Materials and methods

### Patients and controls

MCTD patients (n = 116; mean age ± SD, 59.1 ± 15.9 years, 12 male [11.5%]) were recruited at: Tokyo Metropolitan Tama Medical Center, Sagamihara National Hospital, Kyushu Medical Center, Teikyo University, Nagoya Medical Center, Yokohama City University, Yokohama Minami Kyosai Hospital, Nagasaki Medical Center, and Fukushima Medical University. SLE and SSc patients were also recruited at: Tokyo Metropolitan Tama Medical Center, Sagamihara National Hospital, Kyushu Medical Center, Teikyo University, Nagoya Medical Center, Yokohama City University, Yokohama Minami Kyosai Hospital, and Nagasaki Medical Center. The controls (n = 413; mean age ± SD, 41.4 ± 12.6 years, 62 male [14.0%]) were recruited at Sagamihara Hospital, Kanazawa University, Teikyo University, or by the Pharma SNP Consortium (Tokyo, Japan)^31,32^. The controls and patients were native Japanese. All patients fulfilled the MCTD criteria of Kasukawa et al.^33^ or the criteria for MCTD established by the Research Committee of the Ministry of Health, Labour, and Welfare of Japan^34,35^.

This study was reviewed and approved by the Research Ethics Committees of: Tokyo Metropolitan Tama Medical Center, Sagamihara National Hospital, Kyushu Medical Center, Teikyo University, Nagoya Medical Center, Yokohama City University, Yokohama Minami Kyosai Hospital, Nagasaki Medical Center, Fukushima Medical University, and Tokyo National Hospital. Written informed consent was obtained from all participants. This study was conducted in accordance with the principles expressed in the Declaration of Helsinki.

### Genotyping

*HLA* genotyping of *HLA-DRB1* and *-DQB1* loci was conducted by polymerase chain reaction, using reverse sequence-specific oligonucleotide probes (WAKFlow HLA typing kits, Wakunaga, Akitakata, Japan) with the Bio-Plex system (Bio-Rad, Hercules, CA). Genotyping results for some of the SLE and SSc patients and Japanese controls were previously published^24,25,29^.

### Statistical analysis

Deviation from the Hardy–Weinberg equilibrium was analyzed by Genepop (http://genepop.curtin.edu.au/)^36^. Associations of allele carrier frequencies, genotype frequencies, haplotype carrier frequencies, diplotype frequencies, and amino acid residue carrier frequencies were tested by Fisher’s exact test, using 2 × 2 contingency tables. A multiple logistic regression analysis under the additive model was conducted to determine whether each allele primarily contributes to predisposition to or protection against MCTD. The Bonferroni method was used to correct for multiple comparisons, and corrected *P* (*P*c) values were generated by multiplying *P* values by the number of analyzed alleles or amino acid residues.

## Supplementary Information

Supplementary Information 1.

Supplementary Information 2.

Supplementary Information 3.

Supplementary Information 4.

Supplementary Information 5.

Supplementary Information 6.

## Data Availability

The data that support the findings of this study are not publicly available due to privacy and ethical restrictions. The data are available from the corresponding author upon reasonable request.
