# Supplementary material for: Predisposition of HLA-DRB1*04:01/*15 heterozygous genotypes to Japanese mixed connective tissue disease
Source: Sci Rep. 2022 Jun 15;12:9916. doi: 10.1038/s41598-022-14116-x (PMC9200795; doi:10.1038/s41598-022-14116-x)
Supplement: Supplementary file 1 — Supplementary Information 1. [file 41598_2022_14116_MOESM1_ESM.pdf]

Supplementary Table S1. *DRB1* allele carrier frequency in MCTD patients and controls.

	MCTD (n=116)	Control (n=413)	*P*	OR	*P*c	95%CI
*DRB1*01:01*	10 (8.6)	41 (9.9)	0.8587	0.86	>1	(0.41–1.77)
*DRB1*03:01*	0 (0.0)	2 (0.5)	1.0000	0.71	>1	(0.03–14.82)
*DRB1*04:01*	14 (12.1)	7 (1.7)	$8.66\text{X}10^{-6}$	7.96	0.0003	(3.13–20.24)
*DRB1*04:03*	7 (6.0)	19 (4.6)	0.4766	1.33	>1	(0.55–3.25)
*DRB1*04:04*	2 (1.7)	0 (0.0)	0.0478	18.06	>1	(0.86–378.79)
*DRB1*04:05*	27 (23.3)	87 (21.1)	0.6107	1.14	>1	(0.70–1.86)
*DRB1*04:06*	5 (4.3)	34 (8.2)	0.2257	0.50	>1	(0.19–1.31)
*DRB1*04:07*	0 (0.0)	3 (0.7)	1.0000	0.50	>1	(0.03–9.82)
*DRB1*04:10*	2 (1.7)	14 (3.4)	0.5415	0.50	>1	(0.11–2.23)
*DRB1*07:01*	0 (0.0)	3 (0.7)	1.0000	0.50	>1	(0.03–9.82)
*DRB1*08:02*	11 (9.5)	38 (9.2)	1.0000	1.03	>1	(0.51–2.09)
*DRB1*08:03*	22 (19.0)	61 (14.8)	0.3114	1.35	>1	(0.79–2.31)
*DRB1*08:09*	0 (0.0)	1 (0.2)	1.0000	1.18	>1	(0.05–29.17)
*DRB1*09:01*	43 (37.1)	105 (25.4)	0.0189	1.73	0.5468	(1.12–2.67)
*DRB1*10:01*	0 (0.0)	2 (0.5)	1.0000	0.71	>1	(0.03–14.82)
*DRB1*11:01*	3 (2.6)	21 (5.1)	0.3201	0.50	>1	(0.15–1.69)
*DRB1*12:01*	4 (3.4)	29 (7.0)	0.1955	0.47	>1	(0.16–1.37)
*DRB1*12:02*	1 (0.9)	10 (2.4)	0.4700	0.35	>1	(0.04–2.77)
*DRB1*13:01*	0 (0.0)	5 (1.2)	0.5909	0.32	>1	(0.02–5.81)
*DRB1*13:02*	5 (4.3)	57 (13.8)	0.0032	0.28	0.0929	(0.11–0.72)
*DRB1*14:03*	3 (2.6)	21 (5.1)	0.3201	0.50	>1	(0.15–1.69)
*DRB1*14:04*	0 (0.0)	1 (0.2)	1.0000	1.18	>1	(0.05–29.17)
*DRB1*14:05*	2 (1.7)	14 (3.4)	0.5415	0.50	>1	(0.11–2.23)
*DRB1*14:06*	1 (0.9)	16 (3.9)	0.1383	0.22	>1	(0.03–1.64)
*DRB1*14:07*	0 (0.0)	1 (0.2)	1.0000	1.18	>1	(0.05–29.17)
*DRB1*14:54*	5 (4.3)	28 (6.8)	0.3923	0.62	>1	(0.23–1.64)
*DRB1*15:01*	27 (23.3)	68 (16.5)	0.1008	1.54	>1	(0.93–2.55)
*DRB1*15:02*	20 (17.2)	89 (21.5)	0.3637	0.76	>1	(0.44–1.30)
*DRB1*16:02*	2 (1.7)	5 (1.2)	0.6512	1.43	>1	(0.27–7.48)

Allele carrier frequencies are shown in parentheses (%). Association was tested by Fisher's exact test using 2X2 contingency tables. MCTD: mixed connective tissue disease, OR: odds ratio, CI: confidence interval, *P*c: corrected *P* (*P*c values more than 1 were shown as ">1".).
