# Supplementary material for: Predisposition of HLA-DRB1*04:01/*15 heterozygous genotypes to Japanese mixed connective tissue disease
Source: Sci Rep. 2022 Jun 15;12:9916. doi: 10.1038/s41598-022-14116-x (PMC9200795; doi:10.1038/s41598-022-14116-x)
Supplement: Supplementary file 2 — Supplementary Information 2. [file 41598_2022_14116_MOESM2_ESM.pdf]

Supplementary Table S2. *DQB1* allele carrier frequency in MCTD patients and controls.

	MCTD (n=116)	Control (n=413)	*P*	OR	*P*c	95%CI
*DQB1*02:01*	0 (0.0)	2 (0.5)	1.0000	0.71	>1	(0.03–14.82)
*DQB1*03:01*	23 (19.8)	96 (23.2)	0.5292	0.82	>1	(0.49–1.36)
*DQB1*03:02*	20 (17.2)	83 (20.1)	0.5956	0.83	>1	(0.48–1.42)
*DQB1*03:03*	44 (37.9)	111 (26.9)	0.0279	1.66	0.3903	(1.08–2.57)
*DQB1*04:01*	25 (21.6)	86 (20.8)	0.8975	1.04	>1	(0.63–1.73)
*DQB1*04:02*	8 (6.9)	29 (7.0)	1.0000	0.98	>1	(0.44–2.21)
*DQB1*05:01*	10 (8.6)	44 (10.7)	0.6052	0.79	>1	(0.39–1.63)
*DQB1*05:02*	3 (2.6)	16 (3.9)	0.7774	0.66	>1	(0.19–2.30)
*DQB1*05:03*	6 (5.2)	34 (8.2)	0.3247	0.61	>1	(0.25–1.49)
*DQB1*06:01*	37 (31.9)	144 (34.9)	0.5813	0.87	>1	(0.56–1.36)
*DQB1*06:02*	25 (21.6)	65 (15.7)	0.1616	1.47	>1	(0.88–2.46)
*DQB1*06:03*	0 (0.0)	6 (1.5)	0.3474	0.27	>1	(0.02–4.81)
*DQB1*06:04*	5 (4.3)	50 (12.1)	0.0150	0.33	0.2107	(0.13–0.84)
*DQB1*06:09*	1 (0.9)	6 (1.5)	1.0000	0.59	>1	(0.07–4.95)

Allele carrier frequencies are shown in parentheses (%). Association was tested by Fisher's exact test using 2X2 contingency tables. MCTD: mixed connective tissue disease, OR: odds ratio, 95%CI: confidence interval, *P*c: corrected *P* (*P*c values more than 1 were shown as ">1".).
