# Supplementary material for: Predisposition of HLA-DRB1*04:01/*15 heterozygous genotypes to Japanese mixed connective tissue disease
Source: Sci Rep. 2022 Jun 15;12:9916. doi: 10.1038/s41598-022-14116-x (PMC9200795; doi:10.1038/s41598-022-14116-x)
Supplement: Supplementary file 3 — Supplementary Information 3. [file 41598_2022_14116_MOESM3_ESM.pdf]

SupplementaryTable S3. *HLA-DQB1* genotype frequency in MCTD patients and the healthy controls.

	MCTD (n=116)	Control (n=413)	*P*	OR	95%CI
**03:01 / *06:01*	7 (6.0)	20 (4.8)	0.6335	1.26	(0.52-3.06)
**03:03 / *06:01*	8 (6.9)	19 (4.6)	0.3402	1.54	(0.65-3.60)
**03:01 / *06:02*	5 (4.3)	9 (2.2)	0.2017	2.02	(0.66-6.16)
**03:03 / *06:02*	7 (6.0)	7 (1.7)	0.0178	3.72	(1.28-10.85)

MCTD: mixed connective tissue disease, OR: odds ratio, CI: confidence interval. Genotype frequencies are shown in parentheses (%). Association was tested by Fisher's exact test using 2X2 contingency tables.
