# Supplementary material for: Predisposition of HLA-DRB1*04:01/*15 heterozygous genotypes to Japanese mixed connective tissue disease
Source: Sci Rep. 2022 Jun 15;12:9916. doi: 10.1038/s41598-022-14116-x (PMC9200795; doi:10.1038/s41598-022-14116-x)
Supplement: Supplementary file 4 — Supplementary Information 4. [file 41598_2022_14116_MOESM4_ESM.pdf]

Supplementary Table S4. Conditional logistic regression analysis between the *HLA* alleles in MCTD.

HLA allele	Unconditioned		Conditioned on *DRB1*04:01*		Conditioned on *DQB1*03:01*	
	P	OR (95%CI)	$P_{adjusted}$	$OR_{adjusted}$ (95%CI)	$P_{adjusted}$	$OR_{adjusted}$ (95%CI)
*DRB1*04:01*	$6.93X10^{-5}$	5.92 (2.46–14.21)	NA	NA	$4.49X10^{-6}$	12.46 (4.24–36.62)
*DQB1*03:01*	0.6103	0.89 (0.57–1.39)	0.0106	0.44 (0.24–0.83)	NA	NA
HLA allele	Unconditioned		Conditioned on *DRB1*09:01*		Conditioned on *DQB1*03:03*	
	P	OR (95%CI)	$P_{adjusted}$	$OR_{adjusted}$ (95%CI)	$P_{adjusted}$	$OR_{adjusted}$ (95%CI)
*DRB1*09:01*	0.0096	1.60 (1.12–2.29)	NA	NA	0.8031	1.15 (0.39–3.32)
*DQB1*03:03*	0.0079	1.62 (1.13–2.31)	0.5116	1.43 (0.49–4.11)	NA	NA
HLA allele	Unconditioned		Conditioned on *DRB1*13:02*		Conditioned on *DQB1*06:04*	
	P	OR (95%CI)	$P_{adjusted}$	$OR_{adjusted}$ (95%CI)	$P_{adjusted}$	$OR_{adjusted}$ (95%CI)
*DRB1*13:02*	0.0080	0.29 (0.12–0.72)	NA	NA	0.1522	0.16 (0.01–1.99)
*DQB1*06:04*	0.0191	0.33 (0.13–0.84)	0.5962	2.01 (0.15–26.73)	NA	NA

MCTD: mixed connective tissue disease, OR: odds ratio, CI: confidence interval. *P*, OR, 95%CI, $P_{adjusted}$ and $OR_{adjusted}$ were calculated by logistic regression analysis under the additive model.
