# Supplementary material for: Predisposition of HLA-DRB1*04:01/*15 heterozygous genotypes to Japanese mixed connective tissue disease
Source: Sci Rep. 2022 Jun 15;12:9916. doi: 10.1038/s41598-022-14116-x (PMC9200795; doi:10.1038/s41598-022-14116-x)
Supplement: Supplementary file 5 — Supplementary Information 5. [file 41598_2022_14116_MOESM5_ESM.pdf]

Supplementary Table S5. *DRB1*04:01* allele carrier frequencies in SLE or SSc patients with or without anti-U1RNP Ab.

	Anti-U1RNP Ab(+) SLE (n=142)	Anti-U1RNP Ab(-) SLE (n=201)	*P*	OR	95%CI
*DRB1*04:01*	12 (8.5)	5 (2.5)	0.0204	3.62	(1.25-10.51)
	Anti-U1RNP Ab(+) SSc (n=14)	Anti-U1RNP Ab(-) SSc (n=102)	*P*	OR	95%CI
*DRB1*04:01*	1 (7.1)	1 (1.0)	0.2277	7.77	(0.46-131.84)

SLE: systemic lupus erythematosus, SSc: systemic sclerosis, RNP: ribonucleoprotein, Ab: antibody, OR: odds ratio, CI: confidence interval. Allele carrier frequencies are shown in parentheses (%). Association was tested by Fisher's exact test using 2X2 contingency tables.
