# Supplementary material for: Predisposition of HLA-DRB1*04:01/*15 heterozygous genotypes to Japanese mixed connective tissue disease
Source: Sci Rep. 2022 Jun 15;12:9916. doi: 10.1038/s41598-022-14116-x (PMC9200795; doi:10.1038/s41598-022-14116-x)
Supplement: Supplementary file 6 — Supplementary Information 6. [file 41598_2022_14116_MOESM6_ESM.pdf]

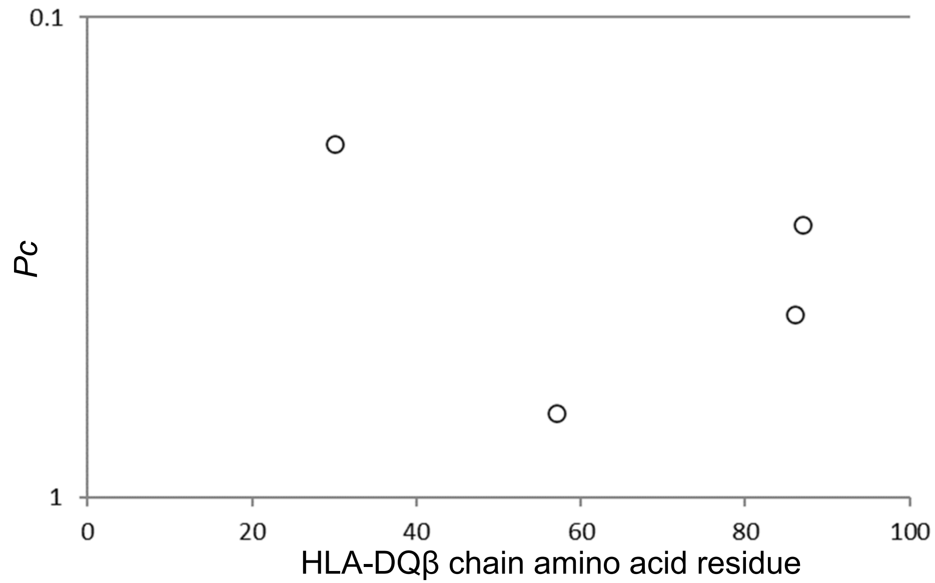


**Supplementary Figure S1. Associations of amino acid residues in the DQβ chain with MCTD.** Associations were established by Fisher's exact test using 2X2 contingency tables. Corrected *P* (*P*c) values were calculated by multiplying the *P* value by the number of amino acid residues tested. Positive associations are indicated by filled circles and negative by open circles. MCTD: mixed connective tissue disease.
